# Rapid Progression of Acute Acalculous Cholecystitis to Gangrenous Cholecystitis in an Initially Non‐Critically Ill Patient: A Case Report

**DOI:** 10.1002/ccr3.73812

**Published:** 2026-10-27

**Authors:** Rami Y. Adam, Sami J. Eldein, Mahmoud M. Mahmoud, Mohamed S. M. Elshikhawoda, Omer Musa, Faris Abdon

**Affiliations:** ^1^ Department of General Surgery Alamal Hospital Khartoum Sudan; ^2^ Department of General Surgery, Faculty of Medicine University of Gadarif Gadarif Sudan; ^3^ Department of Biomedical Sciences College of Medicine, King Faisal University Al Ahsa Saudi Arabia; ^4^ Department of Medical Sciences Orotta College of Medicine and Health Sciences Asmara Eritrea

**Keywords:** acalculous cholecystitis, acute cholecystitis, acute kidney injury, case report, cholecystectomy, gangrene

## Abstract

Acute acalculous cholecystitis (AAC) is not confined to the critically ill and may behave like calculous disease. A 65‐year‐old hypertensive man with localized, stone‐free cholecystitis deteriorated on hospital Day 4 with peritonism, acidosis, and acute kidney injury. Emergency open cholecystectomy confirmed gangrene; deterioration warrants urgent source control.

## Key Clinical Message

Acute acalculous cholecystitis (AAC) can arise outside critical illness, behave like calculous cholecystitis, and still progress rapidly to gangrene. Failure to improve, new generalized abdominal signs, acidosis, oliguria, or renal dysfunction should prompt urgent reassessment and timely source control.

## 1 Introduction

Acute acalculous cholecystitis (AAC) is inflammation of the gallbladder in the absence of gallstones and accounts for approximately 5%–10% of acute cholecystitis in adults [1, 2]. It matters clinically because gangrene, perforation, and empyema are well‐described complications [1, 2]. Mortality in the classic intensive‐care‐associated form may exceed 30%, but that figure reflects the severity of the underlying systemic illness and the associated organ failure at least as much as the gallbladder inflammation itself [2, 3].

AAC is not a single entity. In its classic form, it complicates trauma, sepsis, shock, burns, prolonged fasting, total parenteral nutrition, major surgery, and mechanical ventilation; the gallbladder is often neither markedly distended nor conspicuously thick‐walled, and the underlying critical illness dominates outcome [1, 3, 4]. A second pattern arises outside critical illness and behaves much like calculous cholecystitis, with localized right upper quadrant symptoms in an otherwise ambulatory patient, typically an older man [2, 5]. In this second group, disease of the gallbladder bile and its outflow (inspissated bile or sludge, microlithiasis below the resolution of ultrasonography, or a narrow or functionally obstructed cystic duct) is a more plausible driver than the systemic hypoperfusion invoked in the critically ill [1, 2, 6]. Diagnosis is difficult in both groups because clinical and laboratory findings are nonspecific, and although the Tokyo Guidelines 2018 (TG18) provide diagnostic criteria and severity grading for acute cholecystitis [7], imaging may support the diagnosis without predicting eventual severity [6, 8].

We report AAC in a 65‐year‐old hypertensive man with no trauma, critical illness, or recent major surgery, in whom an initially localized presentation progressed within four hospital days to intraoperatively and histologically confirmed gangrenous cholecystitis, followed by sepsis‐associated acute kidney injury requiring temporary hemodialysis. The instructive element is tempo and sequence rather than rarity: a patient who was not critically ill at presentation became so as a consequence of rapidly progressive gallbladder necrosis, despite an early ultrasound that did not suggest complicated disease. This case report was prepared using the Clinical Case Reports CARE checklist.

## 2 Case History/Examination

A 65‐year‐old man self‐presented to the emergency department of a tertiary care hospital with a several‐day history of progressive right upper quadrant abdominal pain. He was afebrile and reported no preceding diarrhoeal illness or systemic infection. He lived independently at home. His only established comorbidity was hypertension of 2 years' duration, treated with amlodipine 10 mg once daily. He had no history of diabetes mellitus, chronic kidney disease, gallstones or biliary disease, prior abdominal surgery, or drug allergy, and was a lifelong non‐smoker. There was no history of prolonged fasting, dehydration, significant weight loss, trauma, burns, recent myocardial infarction or stroke, immunosuppression, autoimmune disease, known atherosclerotic or peripheral vascular disease, alcohol use, or recreational drug use. Family history was non‐contributory, and the review of systems was otherwise negative.

On arrival, he was alert and in pain. His heart rate was 112 beats/min, blood pressure 135/80 mmHg, respiratory rate 20 breaths/min, oxygen saturation 98% on room air, and axillary temperature 37.1°C. His peripheries were warm, with capillary refill time less than 2 s. Abdominal examination revealed right hypochondrial tenderness with a positive Murphy sign and no guarding, rigidity, or signs of generalized peritonitis. The remainder of the examination was unremarkable. Isolated tachycardia was the only systemic warning sign at presentation.

## 3 Differential Diagnosis, Investigations, and Treatment

Table 1 summarizes the overall sequence of presentation, clinical deterioration, operation, postoperative recovery, and follow‐up.

**TABLE 1:** Timeline of the clinical course.

Time point	Clinical course	Key findings	Management
Presentation/hospital Day 1	Progressive right upper quadrant pain	Tachycardia (112 beats/min); right hypochondrial tenderness; positive Murphy sign; mild leukocytosis (12.5 × 109/L); normal renal and liver biochemistry; ultrasound: distended gallbladder, 5 mm diffuse wall thickening, no stones or sludge	Admitted with a diagnosis of acute acalculous cholecystitis; intravenous fluids, bowel rest, analgesia, and ceftriaxone plus metronidazole
Hospital Days 1–3	Continued inpatient observation	Haemodynamically stable with localized right upper quadrant findings; afebrile; no new peritonism or organ dysfunction; no interval clinical or laboratory change documented that prompted cross‐sectional imaging	Non‐operative management continued with close observation
Hospital Day 4	Abrupt clinical deterioration	Generalized abdominal pain, distension, constipation, upper abdominal guarding; marked leukocytosis (22.0 × 109/L); new renal impairment (creatinine 280 μmol/L); severe metabolic acidosis (pH 7.20, lactate 4.5 mmol/L); oliguria; abdominal radiograph: dilated small‐bowel loops with air–fluid levels	Differential diagnosis included mechanical bowel obstruction, perforated viscus, and gangrenous transformation of acute cholecystitis; resuscitation; decision for emergency exploratory laparotomy without prior CT
Intraoperative	Operative confirmation of disease severity	No mechanical bowel obstruction and no perforated viscus; gangrenous gallbladder without perforation; thick bile; no stones; dense inflammatory adhesions around the gallbladder neck	Primary open midline exploration under general anesthesia (laparoscopy considered but not selected); fundus‐first cholecystectomy; cystic duct stump transfixed with 2–0 polyglactin and covered with omentum; subhepatic closed‐suction drain; ≈120 min; ≈150 mL blood loss
Histopathology	Pathological confirmation	Acute cholecystitis with full‐thickness necrotic mural change; no malignancy	Diagnosis of gangrenous cholecystitis confirmed
Early postoperative period	Critical care course	Sepsis‐associated acute kidney injury (KDIGO stage 3; peak creatinine 450 μmol/L) with severe metabolic acidosis; blood and bile cultures negative; no vasopressors or mechanical ventilation required	Intensive care for 5 days; two hemodialysis sessions; intravenous meropenem plus metronidazole for 7 days with dosing adjusted to renal function and dialysis status; intravenous paracetamol; low‐molecular‐weight heparin
Late postoperative period	Recovery	Renal recovery from postoperative Day 5; creatinine normalized by discharge; gradual clinical improvement	Subhepatic drain removed on postoperative day 3; discharged in good condition after 4 weeks
Follow‐up after discharge	Recovery	Asymptomatic; tolerating a normal diet; normal renal function (creatinine 85 μmol/L)	In‐person surgical review at 3 months; skin sutures removed at the 2‐week review

Abbreviations: CT, computed tomography; KDIGO, kidney disease improving global outcomes.

Initial laboratory testing showed mild leukocytosis with neutrophil predominance, while renal function, liver biochemistry, and electrolytes were within normal limits. C‐reactive protein was mildly elevated (Table 2). No pre‐admission serum creatinine result was available in the records reviewed for this report; the value of 85 μmol/L measured at presentation was therefore the earliest documented result and was taken as the available clinical baseline. Serum amylase and lipase were not measured, as pancreatitis was considered clinically unlikely. Abdominal ultrasonography demonstrated a distended gallbladder with diffuse wall thickening of 5 mm and no gallstones or sludge; the common bile duct, liver, and remaining viscera appeared normal. Because the clinical and sonographic findings were consistent with uncomplicated AAC, computed tomography and hepatobiliary scintigraphy were not pursued at that stage.

**TABLE 2:** Laboratory and physiological parameters at presentation and at clinical deterioration.

Parameter	At presentation (Day 1)	At deterioration (Day 4)	Reference range
White‐cell count (×10^9^/L)	12.5	22.0	4.0–11.0
Neutrophils (×10^9^/L)	9.2	—	2.0–7.5
Hemoglobin (g/L)	140	—	130–170
Platelets (×10^9^/L)	230	—	150–400
C‐reactive protein (mg/L)	25	180	< 5
Procalcitonin (ng/mL)	—	5.2	< 0.5
Creatinine (μmol/L)	85	280	60–110
Urea (mmol/L)	5.5	18.0	2.5–7.5
Sodium (mmol/L)	140	136	135–145
Potassium (mmol/L)	4.1	5.2	3.5–5.0
Chloride (mmol/L)	102	—	98–107
Total bilirubin (μmol/L)	15	30	< 21
Aspartate aminotransferase (U/L)	25	90	< 40
Alanine aminotransferase (U/L)	22	85	< 41
Alkaline phosphatase (U/L)	85	—	40–129
Gamma‐glutamyl transferase (U/L)	35	—	< 61
Arterial pH	—	7.20	7.35–7.45
Bicarbonate (mmol/L)	—	14	22–26
Lactate (mmol/L)	—	4.5	< 2.0
Urine output	Normal	< 0.5 mL/kg/h	≥ 0.5 mL/kg/h

*Note:* A dash (−) denotes a parameter not measured or not documented at that time point. No pre‐admission serum creatinine result was available in the records reviewed for this report; the 85 μmol/L value at presentation was the earliest documented result and was used as the available clinical baseline. Peak creatinine reached 450 μmol/L in the intensive care unit, and creatinine had returned to 85 μmol/L at the 3‐month review.

The initial differential diagnosis included acute calculous cholecystitis, biliary colic, choledocholithiasis, cholangitis, acute pancreatitis, peptic ulcer disease, hepatic pathology, and AAC. Calculous disease became unlikely once ultrasonography showed no gallstones, and choledocholithiasis and cholangitis were discounted because there was no jaundice, the common bile duct was normal, and liver biochemical tests were normal. Pancreatitis and peptic ulcer disease remained considerations, but localizing right upper quadrant signs with gallbladder distension and wall thickening made AAC the leading diagnosis.

The patient was admitted under the general surgical team and initially managed non‐operatively with intravenous fluid resuscitation, bowel rest, analgesia, ondansetron as required, venous thromboembolism prophylaxis, and empirical intravenous ceftriaxone plus metronidazole. This approach was selected because the disease appeared localized, with preserved blood pressure, no peritonism, no organ dysfunction, normal liver biochemical results, and no sonographic evidence of perforation, abscess, or other complication. The plan was close inpatient observation with escalation if the course changed.

During hospital Days 1–3, the patient remained haemodynamically stable, with localized right upper quadrant findings, no fever, and no new peritonism or organ dysfunction. No interval clinical change was documented that would have prompted cross‐sectional imaging, and no interval laboratory results between admission and the day of deterioration were retrievable from the record. On the available documentation, the change on hospital Day 4 was abrupt rather than the endpoint of a visible decline.

On hospital Day 4, his condition deteriorated, with generalized abdominal pain, abdominal distension, constipation, and upper abdominal guarding consistent with evolving peritonitis. Repeat testing showed marked leukocytosis, acute renal impairment, severe metabolic acidosis with hyperlactataemia, and oliguria (Table 2). Plain abdominal radiography showed multiple dilated small‐bowel loops with air‐fluid levels (Figure 1), raising concern for an acute surgical abdomen. The differential now included mechanical small‐bowel obstruction, perforated viscus, and complicated AAC with gangrenous transformation. Gangrenous transformation became the leading diagnosis because of the prior ultrasound‐supported AAC, marked inflammatory progression, metabolic acidosis, oliguria, renal impairment, and deterioration despite antibiotics and supportive care.

**FIGURE 1:**
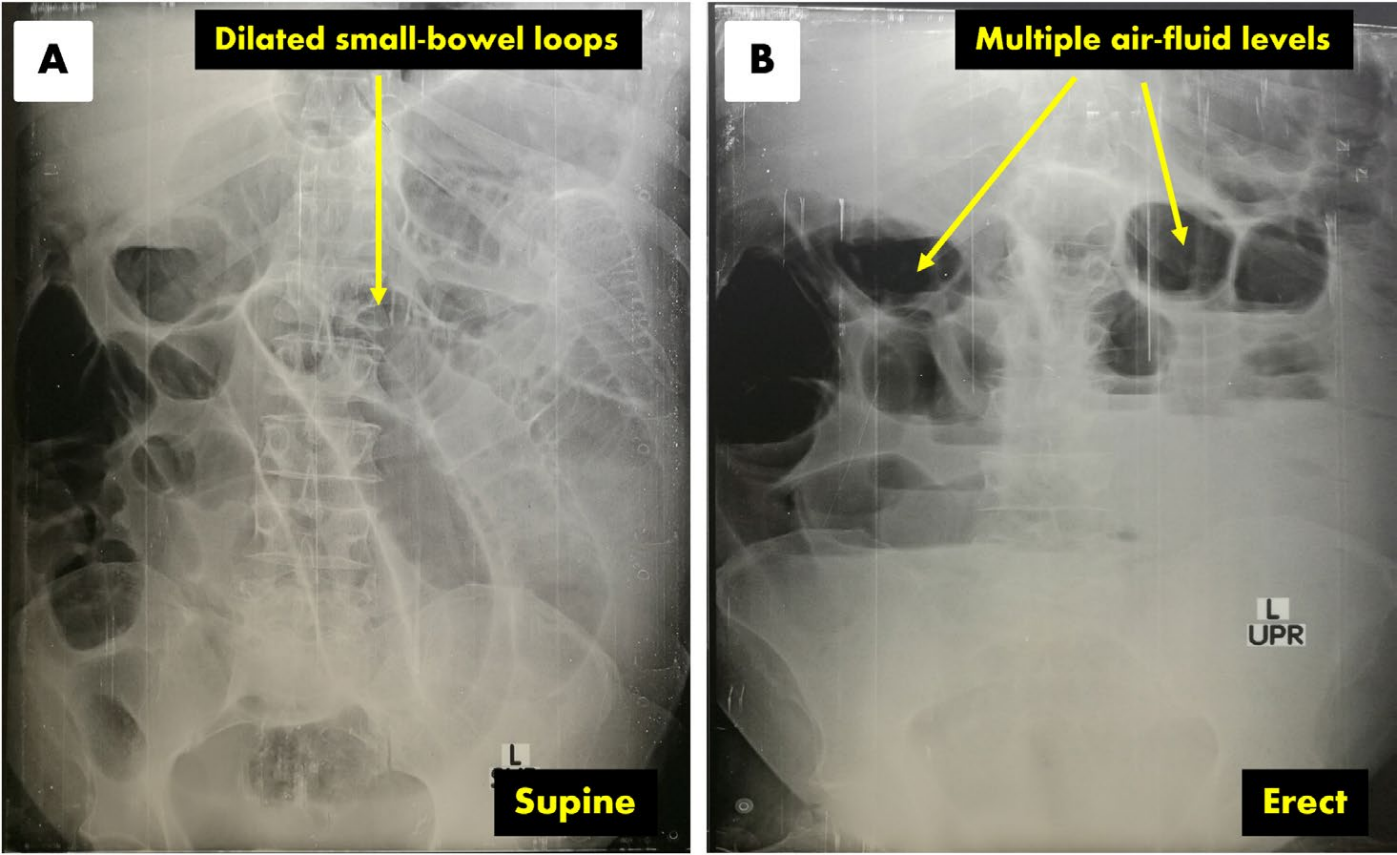
Plain abdominal radiographs obtained after clinical deterioration on hospital Day 4. (A) Supine film showing multiple dilated small‐bowel loops (arrow). (B) Erect film showing multiple air‐fluid levels (arrows).

At deterioration, computed tomography was considered to characterize the acute abdomen further and assess the suspected complicated AAC. However, generalized peritonitis, new organ dysfunction, and severe metabolic derangement made urgent operative source control necessary; the surgical team judged that CT was unlikely to alter the immediate need for exploration and could delay treatment.

Emergency exploratory laparotomy was performed on hospital Day 4 under general endotracheal anesthesia after resuscitation and correction of electrolyte and acid–base derangements. A laparoscopic approach was considered, but primary open midline exploration was chosen because the working differential still included mechanical small‐bowel obstruction and perforated viscus in a distended abdomen with generalized peritonism and severe metabolic derangement; a senior consultant general surgeon operated with the patient supine. Systematic exploration showed no mechanical bowel obstruction and no perforated viscus. The gallbladder was gangrenous but not perforated (Figure 2A). No gallstones were identified, although thick bile was present. Cholecystectomy was technically difficult because of dense inflammatory adhesions around the gallbladder neck. A fundus‐first (dome‐down) dissection was therefore used and carried proximally to the cystic duct, which was divided and secured by transfixion with 2–0 polyglactin (Vicryl) and covered with omentum (Figure 2B); subtotal cholecystectomy was not required. A subhepatic closed‐suction drain was placed. Operative time was approximately 120 min, and estimated blood loss was 150 mL. There was no documented intraoperative bile duct or vascular injury. Intraoperative blood and bile cultures were negative. Histopathology confirmed acute cholecystitis with full‐thickness necrotic mural change, consistent with gangrenous cholecystitis. There was no evidence of malignancy.

**FIGURE 2:**
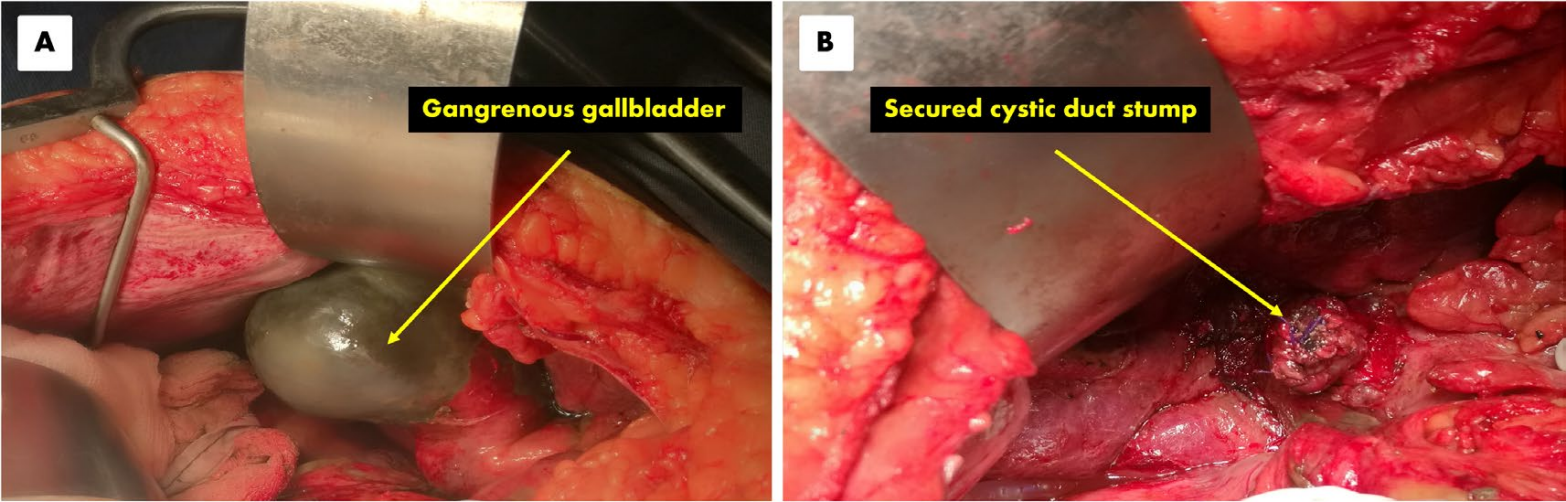
Intraoperative findings in gangrenous acute acalculous cholecystitis. (A) Distended, discolored gangrenous gallbladder identified at laparotomy (arrow). (B) Operative field after fundus‐first cholecystectomy showing the cystic duct stump secured by transfixion (arrow) within markedly inflamed surrounding tissue.

## 4 Outcome and Follow‐Up

Postoperatively, the patient was managed in the intensive care unit for sepsis‐associated acute kidney injury, staged as Kidney Disease: Improving Global Outcomes (KDIGO) Stage 3 [9] with a peak creatinine of 450 μmol/L, and for severe metabolic acidosis related to the intra‐abdominal source. He did not require vasopressor support or mechanical ventilation. He required two sessions of temporary hemodialysis. Renal function began to recover from the fifth postoperative day, with restoration of spontaneous urine output and improvement in acid–base status. Serum creatinine returned to baseline by discharge.

The intensive care stay lasted 5 days, and the total hospital stay was 4 weeks. The documented postoperative antimicrobial regimen was intravenous meropenem plus metronidazole for 7 days. A carbapenem‐based regimen was selected empirically because severe intra‐abdominal sepsis with organ dysfunction had developed despite ceftriaxone and metronidazole, and the treating team adjusted dosing according to renal function and dialysis status. Blood and bile cultures were negative, so microbiologically guided de‐escalation was not possible. The available records did not permit reliable reconstruction of every individual dose adjustment during rapidly changing renal function, and no doses have been inferred retrospectively. Additional postoperative care comprised intravenous paracetamol for analgesia, renal‐supportive care, and low‐molecular‐weight heparin for venous thromboembolism prophylaxis. The subhepatic drain was removed on postoperative Day 3 after minimal output, and skin sutures were removed at the 2‐week review. The patient adhered to treatment and follow‐up. At the 3‐months in‐person follow‐up in the surgical outpatient clinic, he remained asymptomatic, had no recurrent abdominal pain, tolerated a normal diet, and had normal renal function (creatinine 85 μmol/L, equal to the value recorded at presentation). No further abdominal imaging was required because he was asymptomatic. No standardized patient‐reported outcome measure was used.

## 5 Patient Perspective

At follow‐up, the patient reported that he had recovered well, was eating normally, and was relieved that the abdominal pain had resolved. He expressed gratitude for the urgent surgical and intensive‐care treatment. No direct patient quotation was used.

## 6 Discussion

Separating the two clinical patterns of AAC is central to interpreting this case. In critically ill patients, AAC arises against a background of hypoperfusion, vasoactive support, fasting, and systemic inflammation; the gallbladder may be neither distended nor conspicuously thick‐walled, and the underlying illness largely determines outcome [1, 3, 10]. Our patient was ambulatory and self‐presenting, haemodynamically stable, and had a distended, uniformly thickened gallbladder with a positive Murphy sign; this phenotype reflects the second, calculous‐like pattern of AAC [2, 5]. The contribution of this report is therefore not the fact of a non‐critical presentation but the sequence: calculous‐like AAC at presentation, gangrenous transformation within 4 days, and secondary organ dysfunction that rendered a previously well patient critically ill.

The most parsimonious explanation for that course is disease of the gallbladder bile and its outflow rather than systemic hypoperfusion. Thick, inspissated bile was found at operation in the absence of stones, and impaired emptying, biliary sludge, occult microlithiasis, or a narrow or functionally obstructed cystic duct can each reproduce the final common pathway of an impacted stone [1, 2, 6]. None of these was demonstrated here, and none can be excluded retrospectively, since ultrasonography does not reliably detect microlithiasis, and neither stimulated scintigraphy nor bile microscopy was performed.

Where outflow is impaired, the consequences are mechanical before they are ischaemic. Continued secretion into an obstructed gallbladder raises intraluminal pressure, which first exceeds venous and lymphatic outflow pressure and produces mural congestion and oedema, and then reduces capillary perfusion of the wall itself [1, 3]. The gallbladder tolerates this poorly, because it depends largely on a single cystic artery with little collateral inflow beyond small vessels crossing the cystic plate, and because distension is greatest at the fundus, the most distal territory of that supply and the usual site at which necrosis and perforation begin [3, 11]. Inflammatory mediator release, ischaemia‐reperfusion injury, and secondary bacterial invasion of a devitalised wall complete the transition from mural inflammation to transmural necrosis [1, 2]. Compressed into a few days, a sequence of this kind is consistent with the tempo observed in our patient.

A central practical lesson is that first‐line imaging can establish AAC without defining its severity. Ultrasonography is the appropriate initial test, but its sensitivity in AAC is variable and severe disease may be underestimated [8, 10, 12]. Findings that should raise concern for complicated or gangrenous disease include irregular or striated wall thickening, intraluminal membranes or sloughed mucosa, pericholecystic fluid or abscess, marked distension and, on contrast‐enhanced CT, absent or irregular mural enhancement [13, 14, 15]. None of these were present at admission in our patient, and no new warning sign was documented during hospital Days 1–3; the decisive change was clinical rather than sonographic. With hindsight, the safer general rule is not to wait for organ dysfunction: in AAC that has not clearly improved after 48–72 h of antibiotics and supportive care, repeat ultrasonography or contrast‐enhanced CT is reasonable, provided imaging does not delay source control once peritonitis is established [10, 15].

Preoperative recognition of gangrenous cholecystitis is frequently missed [16, 17]. Reported associations include older age, male sex, diabetes, hypertension, marked leukocytosis, gallbladder wall thickening, pericholecystic fluid, and greater gallbladder distension [11, 16, 17, 18, 19]. Our patient had older age, male sex, hypertension, leukocytosis, and wall thickening, but not diabetes. These are statistical associations rather than demonstrated mechanisms. Although hypertension is reported more often among patients with gangrenous cholecystitis in observational cohorts [18, 19] he had no clinically apparent atherosclerotic disease and no vascular assessment was undertaken, so atherosclerosis should not be advanced as the cause of gangrenous transformation here; in the calculous‐like form of AAC, it is likely an uncommon primary mechanism. The practical implication is simpler: when inflammatory gallbladder disease deteriorates clinically, gangrene should be suspected even if earlier imaging was unremarkable.

From a management perspective, failure to improve, or the emergence of diffuse abdominal signs, acidosis, oliguria, or organ dysfunction should prompt urgent reassessment and a low threshold for source control; in critically ill or non‐operative candidates, antibiotics with percutaneous cholecystostomy remain important alternatives [20]. The operative approach here deserves comment, because TG18 positions laparoscopic cholecystectomy, rather than laparotomy, as the standard operative approach when it is feasible [21], and laparoscopy carries lower postoperative morbidity than open surgery even in gangrenous disease [11]. Two considerations made primary open exploration the safer option on Day 4. First, the working differential still included mechanical small‐bowel obstruction and perforated viscus, and the abdomen was distended, which limits both working space and the diagnostic yield of laparoscopy. Second, severe metabolic acidosis with hyperlactataemia and evolving acute kidney injury made prolonged pneumoperitoneum unattractive. Open exploration allowed assessment of the whole abdomen and source control without delay. This was a case‐specific judgment, not a claim that laparotomy is preferable in AAC; in a stable patient with disease confined to the gallbladder, a laparoscopic start with a low threshold for conversion would be appropriate.

Dense inflammation around the gallbladder neck also dictated the dissection technique. Fundus‐first (dome‐down) dissection and subtotal fenestrating or reconstituting cholecystectomy are both endorsed bailout strategies when the critical view of safety cannot be obtained safely [21, 22]. In this patient, the fundus‐first plane could be developed as far as the cystic duct, so subtotal cholecystectomy was unnecessary. Where the duct is oedematous and friable, clips are unreliable; a transfixion suture or endoloop is preferable, and drainage of the subhepatic space is prudent.

Antimicrobial management illustrates two stewardship points. Escalation to a carbapenem was defensible in culture‐negative severe intra‐abdominal sepsis that had progressed despite a third‐generation cephalosporin and metronidazole; the metronidazole, however, added no useful anaerobic cover to meropenem and reflected continuation of the empirical regimen rather than a deliberate choice. Once source control has been achieved, a fixed short course of about 4 days is as effective in complicated intra‐abdominal infection as therapy continued until the features of sepsis resolve [23], so 7 days was longer than strictly necessary.

The principal strength of this report is its clear temporal documentation of progression from sonographically diagnosed AAC to intraoperatively and histologically confirmed gangrenous cholecystitis, supported by a clinical timeline, laboratory trends, radiographic and intraoperative images, and follow‐up demonstrating clinical and renal recovery. It is relevant to emergency surgery, acute care, radiology, and critical care because it links initial diagnostic uncertainty with the later need for time‐critical source control.

The report has limitations. It is a single case and cannot establish a causal mechanism for gangrenous transformation. Occult microlithiasis, biliary sludge, and a cystic‐duct factor could not be excluded, and no vascular assessment was performed. CT and hepatobiliary scintigraphy were not obtained, so multimodality radiological comparison is unavailable. No pre‐admission creatinine was available, no interval laboratory results between admission and hospital Day 4 were retrievable, and the retrospective record did not permit reconstruction of each antimicrobial dose adjustment during evolving acute kidney injury and hemodialysis. No formal quality‐of‐life measure was used, and follow‐up was limited to 3 months. These limitations do not alter the central clinical message: AAC can begin outside the traditional critically ill setting, and clinical deterioration should prompt urgent reassessment even when initial ultrasound findings appear unalarming.

## 7 Conclusion

Acute acalculous cholecystitis may present in patients who are not critically ill at the outset, may behave like calculous cholecystitis, and can still progress rapidly to gangrenous cholecystitis, occasionally precipitating secondary organ dysfunction such as sepsis‐associated acute kidney injury. An initial ultrasound can support the diagnosis but may not reflect the eventual severity. Failure to improve, new generalized abdominal signs, marked inflammatory progression, metabolic acidosis, oliguria, or renal dysfunction should prompt urgent reassessment and timely source control.

## Author Contributions

**Rami Y. Adam:** conceptualization, data curation, investigation, supervision, writing – original draft, writing – review and editing. **Sami J. Eldein:** conceptualization, investigation, supervision, writing – review and editing, data curation. **Mahmoud M. Mahmoud:** conceptualization, data curation, investigation, supervision, writing – review and editing. **Mohamed S. M. Elshikhawoda:** methodology, resources, validation, visualization, writing – review and editing. **Omer Musa:** methodology, resources, validation, visualization, writing – review and editing. **Faris Abdon:** conceptualization, methodology, resources, writing – original draft, writing – review and editing.

## Funding

The authors have nothing to report.

## Ethics Statement

This case report was conducted in accordance with the Declaration of Helsinki. Institutional ethics approval was not required for a single, de‐identified case report under local institutional policy.

## Consent

Written informed consent was obtained from the patient for publication of this case report and any accompanying images. The patient has been fully anonymised.

## Conflicts of Interest

The authors declare no conflicts of interest.

## Data Availability

The data that support the findings of this study are available from the corresponding author upon reasonable request.
